# *In Vivo* Measurement of Oxygenation Changes after Stroke Using Susceptibility Weighted Imaging Filtered Phase Data

**DOI:** 10.1371/journal.pone.0063013

**Published:** 2013-05-13

**Authors:** Meng Li, Jiani Hu, Yanwei Miao, Huicong Shen, Dingbo Tao, Zhihong Yang, Qinghang Li, Stephanie Y. Xuan, Waqar Raza, Sadeer Alzubaidi, E. Mark Haacke

**Affiliations:** 1 Department of Radiology, Wayne State University, Detroit, Michigan, United States of America; 2 Department of Radiology, The First Affiliated Hospital, Dalian Medical University, Dalian, Liaoning, China; 3 Department of Radiology, Beijing Tiantan Hospital, Capital Medical University, Beijing Neurosurgical Institute, Beijing, China; 4 Department of Neurology, The First Affiliated Hospital, Dalian Medical University, Dalian, Liaoning, China; 5 Department of Radiology, Third Hospital of Xingtai, Xingtai, Hebei, China; 6 Department of Neurological Surgery, Wayne State University, Detroit, Michigan, United States of America; 7 University of Toronto, Faculty of Arts & Science, Toronto, Ontario, Canada; University of Minnesota, United States of America

## Abstract

**Background and Purpose:**

Cerebral blood oxygenation level is critical for following the evolution of stroke patients. The purpose of this study was to investigate the feasibility of measuring changes in blood oxygen levels for patients with acute stroke using SWI and to compare these changes with the patient's recovery over time.

**Materials and Methods:**

A total 30 MRI scans was performed on 10 acute ischemic stroke patients. Every patient was followed at three time points: less than 24 hours; 2–3 weeks after stroke and 2 months after stroke. Both MRI scan and NIH stroke scale (NIHSS) were acquired for each patient at all three time points. Oxygen saturation changes were derived from phase values differences (Δφ) measured over 10 veins from each hemisphere for all 10 patients over 3 time points. The correlation of oxygen saturation and NIHSS was further evaluated.

**Results:**

The stroke affected side of the brain showed moderate (r = −0.62) to strong (r = −0.70) correlation between the oxygenation change and NIHSS change. The oxygen saturation change from the normal side of the brain had essentially no association with recovery (r = −0.02 and−0.31). The results suggest that increases in oxygen saturation correspond to improved outcome and reductions in oxygen saturation correspond to worse outcome.

**Conclusion:**

High resolution SWI provided a novel method to measure changes in oxygenation change of the human brain *in vivo*. By using the phase values from the veins, both spatial and temporal information can be found that relates to patient outcome post stroke.

## Introduction

Hemoglobin exists in the human body in two distinct states, oxyhemoglobin and deoxyhemoglobin, which have different magnetic susceptibilities. Oxyhemoglobin is the state where oxygen is bound to the hemoglobin molecule and exhibits only a very small diamagnetic susceptibility relative to surrounding brain tissue. Deoxyhemoglobin is the state where oxygen is not bound to the hemoglobin and exhibits paramagnetic properties relative to surrounding brain tissue. Consequently, changes in oxygen saturation levels will lead to a phase difference between veins and the surrounding tissue [1]. By comparing changes in venous phase over multiple time points, it is possible to estimate the changes in oxygen saturation. Ischemic stroke is caused by the blocking of blood vessels, reduced oxygen delivery and increased levels of deoxyhemoglobin. If the delivery of blood is interrupted for too long, then the affected brain tissue will cease to function and ultimately die. Therefore, monitoring levels of oxygen saturation is key to determining tissue viability post stroke.

Susceptibility Weighted Imaging (SWI) is a high resolution 3D phase enhanced gradient echo method with full flow compensation in all three directions [2]. In SWI, susceptibility differences of neighboring tissues are exploited to increase the contrast of MR images, and can therefore aid in identifying tissue properties and states in both magnitude and phase images. SWI is sensitive to the presence of venous blood, hemorrhage and iron storage. By measuring the phase difference between local small veins and surrounding tissue, the venous oxygenation information can be obtained. In particular, SWI filtered phase can be used to measure changes in blood oxygen content. In acute stroke, SWI makes it possible to detect the microbleeding [3] in the infarction and intravascular clot in the thrombosed vessel [4]. Moreover, SWI can be used to evaluate the oxygen extraction fraction (OEF) and the cerebral metabolic rate of oxygen in the acute stroke due to an increased ratio of deoxyhemoglobin to oxyhemoglobin in the affected region. OEF is considered a critical marker of tissue viability and can be used to directly assess oxygen metabolism in ischemic tissue [5]. The purpose of this study is to investigate the feasibility of measuring blood oxygen levels for patients with stroke using phase information to observe the correlation of changes in oxygen saturation over time with patient recovery.

## Materials and Methods

Changes in the local magnetic field, ΔB, determine spatial variation in the phase both inside and outside the structure. These field changes give rise to a twofold effect: (i) dephasing of the signal outside the structure and in the pixel containing the structure leading to a reduction of T_2_^*^ and (ii) the presence of measurable phase [6]–[8]. The SWI magnitude images are used to enhance the sensitivity to both T2* and phase changes to reveal abnormal looking veins. However, assigning an oxygen saturation to these images is difficult. On the other hand, the SWI filtered phase images can be used as a direct measure of the susceptibility from the vessels and are a representation of local oxygen saturation [9]–[11]. To appreciate this, consider first the phase behavior as a function of position “r” as given by: 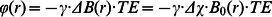
(1) where γ is the gyro-magnetic ratio of the proton (2.678×10^8^ rad/s/T), ΔB is the change in magnetic field between tissues, B_0_ is the static magnetic field, TE is the echo time and Δχ is the local magnetic susceptibility change between tissues.

**Figure 1:**
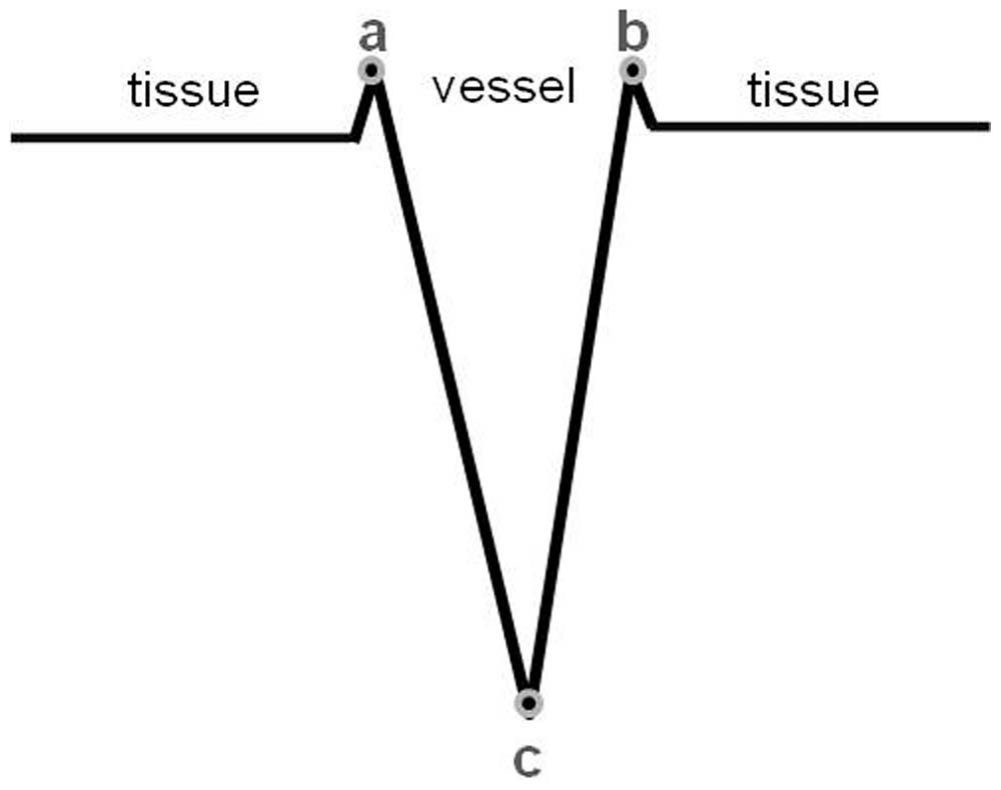
Phase profile showing the susceptibility change from the tissue and vessel. We measure Δφ_a_, Δφ_b_ and Δφ_c_, so the average phase change cross the vessel 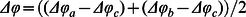.

Assuming the blood vessel is an infinite cylinder model [11], the phase difference Δφ between the blood in the vessel and the surrounding tissue can be expressed as: 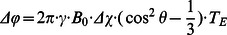
(2) where θ represents the angle between the blood vessel and the static magnetic field B_0_. The susceptibility difference Δχ is expressed as:
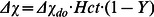
(3) where Δχ_do_ [12] is the change in blood susceptibility per unit hematocrit between fully deoxygenated and fully oxygenated blood, and has been measured to be 1.8×10^−7^. The average oxygen saturation level *Y* is 0.55 [11]. *Hct* is the percent of red blood cells in a given volume of whole blood. The relationship between the oxygenation change ΔY over the time and oxygen saturation is expressed as:
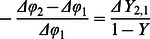
(4) where 1 and 2 refer to the two MRI scans at different time points. The measured phase difference Δφ_1_ and Δφ_2_ are from the same vessel from the images acquired at these two time points. How to measure Δφ is illustrated in Figure 1. The changes of oxygenation ΔY_2,1_ can be obtained from Equation (4). ΔY_2,1_ is independent of blood vessel orientation due to cancellation of the geometry dependent term (3cos^2^θ−1).

### Subjects

A total of 10 acute ischemic stroke patients aged from 20 to 74 years were recruited in this study. There were 6 males and 4 females, the mean and standard deviation of the age were 56.4 years and 18.0 years, respectively. All patients were not treated with thrombolysis during this study. The NIH stroke scale (NIHSS) was assessed by an experienced neurologist. Both MRI scans and NIHSS measures were acquired for each patient at three time points: 1) within the first 24 hours; 2) 2–3 weeks after stroke; and 3) 2 months after stroke. Informed consent was obtained from all subjects, and all protocols were approved by the local institutional review board.

### MR imaging

All MR imaging was acquired on a 1.5T magnet (GE Signa HD1.5T) with an eight-channel head coil. The SWI sequence used was a fully velocity-compensated, three-dimensional, gradient-echo sequence, with the following parameters: TE/TR = 40/50 ms, FA = 20°, pixel bandwidth is 122 Hz/pixel, slice thickness = 2 mm, field-of-view (FOV) = 256×256, and an acquisition matrix = 512×512. After all sequences were acquired, the SWI source data were post-processed using SPIN (signal processing in NMR) software (Detroit, Michigan). High pass filtering was applied using a central matrix size of 64×64 [2].

Other MRI sequences used included: FLAIR, T2-weighted imaging (T2WI), T1-weighted imaging (T1WI) and diffusion weighted imaging (DWI). The combination of DWI, FLAIR and SWI was used to define the location and extent of the stroke lesion. Data were collected with the parameters for FLAIR: TR/TE = 8602/123 ms, FA = 90°, FOV = 288 mm×192 mm, BW = 122 Hz/pixel and TH = 6 mm with a 1 mm gap; for T2WI: TR/TE = 3000/104 ms, FA = 90°, FOV = 288 mm×192 mm, BW = 122 Hz/pixel and TH = 6 mm with a 7.5 mm gap; for T1WI: TR/TE = 400/15 ms, FA = 90°, FOV = 288 mm×192 mm, BW = 61.05 Hz/pixel and TH = 6 mm with a 1 mm gap; and for DWI: TR/TE = 6000/82 ms, FA = 90^o^, FOV = 128 mm×128 mm, BW = 1953 Hz/pixel, TH = 6 mm with a 1 mm gap and b = 0, 1000 s/cm^2^.

To keep the consistency of the patient's head position in the three time points, the patient's head was adjusted to make the line connected with anterior commissure and posterior commissure (AC-PC line) perpendicular to the static magnetic field (B_0_) based on the localizer image on the sagittal view. Further, we kept the same slice thickness and interval at every scan for each patient, and the same slice location based on the AC-PC line.

### Vessel phase measurements

**Figure 2:**
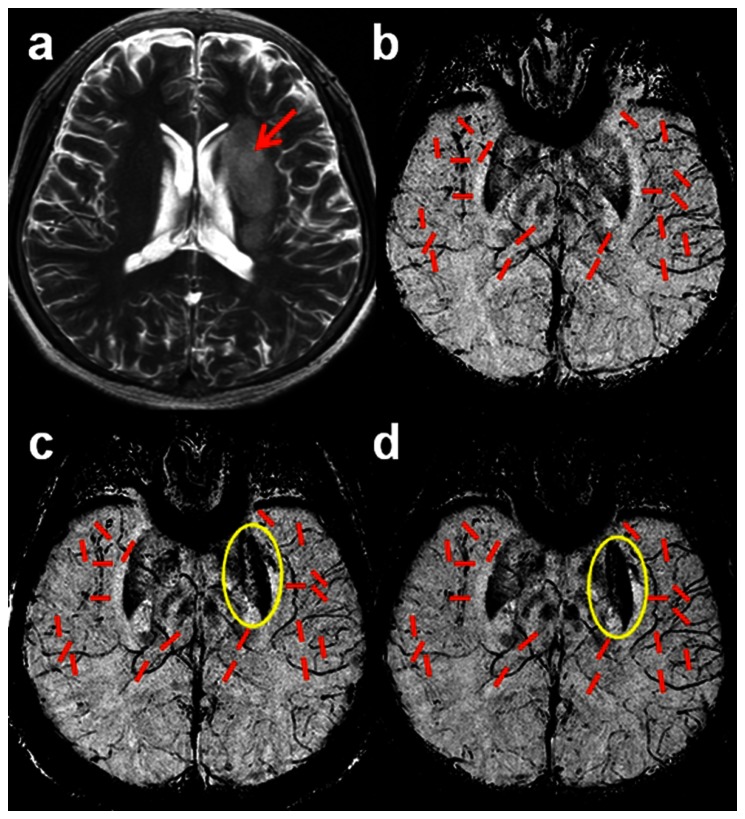
Vessel selection for a stroke patient. a) maximum intensity projection over 3 slices of T_2_ weighted imaging shows stroke affected area (red arrow). b), c) and d) minimum intensity projections over 20 slices of SWI processed phase images showing the locations of the 10 measured vessels (red lines) for each hemisphere at (b)<24 hours, (c) 2 to 3 weeks after stroke, and (d) 2 months after the onset of stroke. SWI shows hypointense signal on the stroke side of the brain (c,d), and also a hemorrhage (yellow circle) in the left putamen in the second and third scans (c,d).

The criteria of the vessel selection were: 1) each vessel showed clearly in all three MRI scans; 2) no vessel close to the skull or sinuses was chosen (to avoid air/tissue interface artifacts); 3) 20 vessels were chosen for each patient, with 10 on the ipsilateral side and 10 on the contralateral side. Figure 2 shows the vessel selection for one patient. Therefore, a total of 600 veins was valuated from all 10 patients to obtain oxygen saturation level changes.

**Figure 3:**
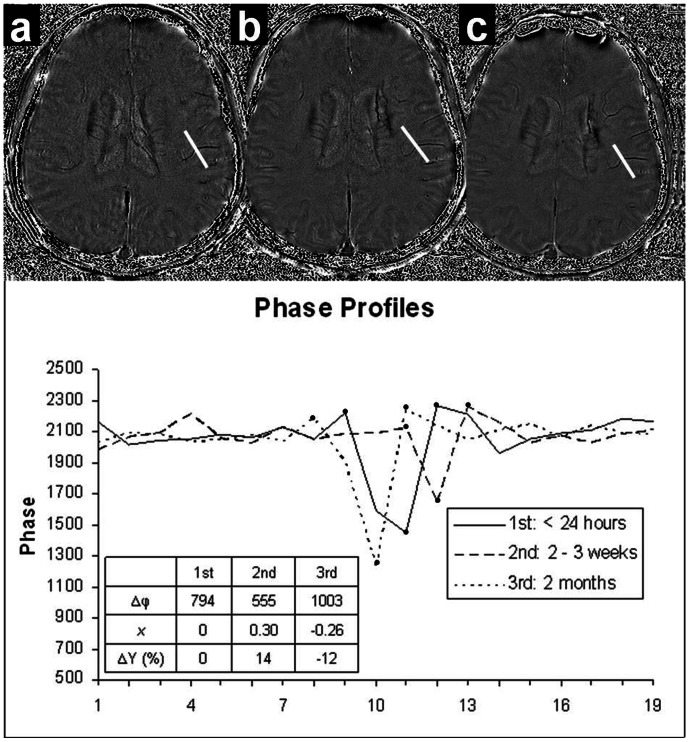
Phase profiles over the SWI filtered phase images corresponding to the three MRI scans (a)<24 hours, (b) 2–3 weeks after stroke, and (c) 2 months after the onset of stroke.

The procedures used to obtain Δφ_1_, Δφ_2_ and Δφ_3_ are illustrated in figure 3. For a particular vessel, each phase profile is obtained along a line cutting across the vessel as indicated in Figures 3a–c. The phase difference is found by averaging the peak values for both sides of the vessel wall and subtracting the minimum phase of the vessel (Figure 1). The profile corresponding to the first time SWI scan (<24 hours) is designated Δφ_1_ and the remaining two time point profiles are designated by Δφ_2_ and Δφ_3_. These values are then used in equation (4) to derive the oxygenation change ΔY. The comprehensive oxygenation changes of each side of the brain are then obtained by averaging over the ten measured vessels. We calculated oxygenation changes for two time intervals: 1) using Δφ_1_ as the baseline phase data, and with Δφ_2_ to get oxygenation change ΔY_2,1_ from the first and second MRI scans; and 2) using Δφ_2_ as the baseline phase data along with Δφ_3_ to get the oxygenation change ΔY_3,2_ from the second MRI scan to the third MRI scan.

### Correlating oxygen saturation changes with NIHSS

To evaluate NIHSS change, the later NIHSSs were subtracted from the previous ones to obtain ΔNIHSS_2,1_ and ΔNIHSS_3,2_. A positive change indicates a worsening of their condition and a negative change indicates an improvement in their condition.

## Results

The gender, age, and three NIHSS scores are given in Table 1. We found that 8 patients had various degrees of recovery with the NIHSS values changing from−2 to−8 at the second time point. There were two patients who got worse with the scores increasing by 2 and 3. However, the third score showed that after two months all patients showed some improvement ranging from−2 to−10 compared to their first ones.

**Table 1:** List of the ten patient's age and their assessments of NIHSS at the three MRI scans.

Subject	Gender	Age	NIHSS	NIHSS	NIHSS	NIHSS changes	NIHSS changes
			(1^st^)	(2^nd^)	(3^rd^)	(2^nd^–1^st^)	(3^rd^–2^nd^)
1	F	61	16	14	9	−2	−5
2	M	59	8	10	1	2	−9
3	M	54	7	1	0	−6	−1
4	M	20	2	0	0	−2	0
5	F	62	17	15	13	−2	−2
6	F	70	8	4	1	−4	−3
7	M	74	10	5	2	−5	−3
8	F	72	5	8	2	3	−6
9	M	29	11	9	9	−2	0
10	M	63	23	15	13	−8	−2
Mean		56.40	10.70	8.10	5.00	−2.60	−3.10
Standard Deviation		18.02	6.29	5.55	5.37	3.37	2.85

List of the ten patient's age and their assessments of NIHSS at the three MRI scans: 1^st^ is<24 hours, 2^nd^ is 2–3 weeks after stroke, and 3^rd^ is 2 months after the onset of stroke.

**Figure 4:**
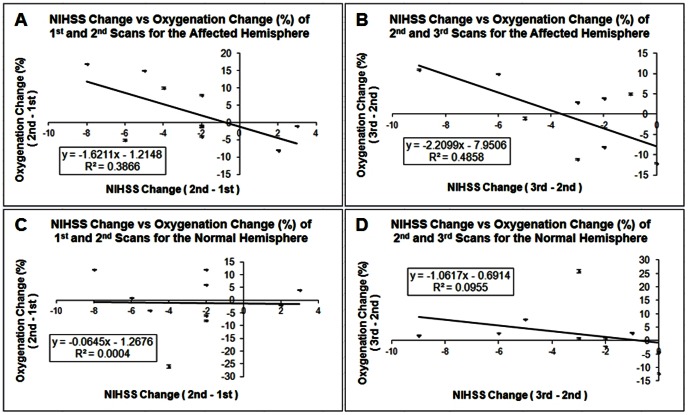
Oxygenation changes vs. NIHSS changes scatter plots with error bar of both hemispheres for ten stroke patients. A) 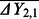 vs. ΔNIHSS2,1 for the stroke hemisphere; B) 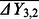 vs. ΔNIHSS3,2 for the stroke hemisphere; C) 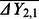 vs. ΔNIHSS2,1 for the non-stroke hemisphere; D) 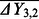 vs. ΔNIHSS3,2 for the non-stroke hemisphere. Here 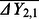 and 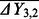 are the average over 10 vessels for each hemisphere.

The results of Figure 4 show that the oxygenation change is negatively correlated with the NIHSS change. The correlation between the average oxygenation change and the NIHSS change for the second and the first imaging time points is shown in Figure 4a and 4c. The coefficients of determination R^2^ were 0.3866 for the affected side of the brain (4a), and 0.0004 for the normal side of brain (4c). The correlation coefficients from the ten patients were−0.62 (4a) and−0.02 (4c) for the stroke hemisphere and the non-stroke hemisphere respectively. Figures 4b and 4d are the scatterplots for the oxygenation change and NIHSS change for the third and the second imaging time points. The coefficients of determination R^2^ were 0.4858 and 0.0955 for the affected side of the brain (4b) and the normal side of brain (4d) respectively. The correlation coefficients from the ten patients were−0.70 (4b) for the stroke hemisphere and−0.31 (4d) for the non-stroke hemisphere. The results indicate that the affected side of the brain showed moderate to strong correlation between the oxygenation change and NIHSS change. However, the normal side of the brain showed weak or no association between these two variables.

Analysis of variance (ANOVA) based on linear regression was applied to test the significance of the overall regression. The confidence level was set as 95%. The results are shown in Table 2. For the regression model of the data acquired from the second and the first scan, the p-values were 0.05 and 0.95 for the affected side of brain and normal side of brain respectively. ANOVA analysis for the data acquired from the third and the second scan, the p-values were 0.02 and 0.38 for the stroke hemisphere and the non-stroke hemisphere respectively. The correlation between oxygenation changes and NIHSS changes has significance for the period from the second to the third scan.

**Table 2:** ANOVA test results of four regression models for both hemispheres.

Scan timepoint	ROI		df	SS	MS	F	Significance F
2^nd^ scan – 1^st^ scan	affected	Regression	1	269.10	269.10	5.04	0.05
	hemisphere	Residual	8	426.90	53.36		
		Total	9	696.00			
							
	normal	Regression	1	0.43	0.43	0.00	0.95
	hemisphere	Residual	8	1124.47	140.56		
		Total	9	1124.9			
							
3^rd^ scan – 2^nd^ scan	affected	Regression	1	356.01	356.01	7.56	0.02
	hemisphere	Residual	8	376.89			
		Total	9	732.9			
							
	normal	Regression	1	82.18	82.18	0.84	0.38
	hemispheres	Residual	8	778.22	97.28		
		Total	9	860.4			

ANOVA test results of four regression models which show in Figure 4 for both hemispheres. The stroke hemisphere show the significant correlation between the oxygenation change vs. the NIHSS change for the time period of the third and the second MRI scan.

## Discussion

Oxygenation level is a vital parameter to monitor in intensive care units for stoke patients and a number of other diseases such as traumatic brain injury. Positron emission tomography (PET) is available for clinical measurement of oxygenation; however, given its invasive nature and low spatial resolution, it is still not routinely utilized in critical care. SWI is a non-invasive, rapid MRI technique that can provide high spatial resolution for imaging oxygen saturation changes. Preliminary results from this study demonstrate that SWI can provide an *in vivo* estimate of blood oxygen saturation levels for patients with stroke and that decreases in phase (increases in oxygen saturation) correlate with patient outcome. Compared to traditional perfusion methods such as dynamic-susceptibility contrast-enhanced MR imaging, SWI provides higher resolution images and estimates of oxygenation changes without the injection of a contrast agent and can be used to assess indirectly perfusion deficits and the penumbra in acute stroke [5].

Ischemic stroke occurs as the result of obstruction within a blood vessel that supplies blood to the brain, causing a deficiency in blood flow (ischemia). During an ischemic stroke, the brain initiates a series of events that may result in delayed damage to brain cells [13]. In this study, there were major changes seen in the oxygenation changes on the ipsilateral (stroke) side but there were few effects seen on the contralateral (normal) side. We postulate that an increase in the brain oxygenation level in the stroke region could result in an increase of the regional oxygen extraction fraction (OEF) [14] and improved brain tissue viability. Our study demonstrated that after acute ischemic stroke, the clinical outcomes depended on the oxygen saturation level in the stroke affected area.

The oxygenation for each hemisphere is from the average over the measurements from 10 vessels. The vessels were chosen as close as possible to the stroke region in the affected hemisphere. Different veins drain blood into different regions of the brain, while only specific regions are affected by the stroke. So even within the same hemisphere, vessel-wise comparisons demonstrated that temporal changes of oxygenation were vessel and regional specific.

SWI filtered phase imaging not only provides a non-invasive and safe measurement of oxygen saturation changes but also provides the most sensitive means to detect hemorrhage after stroke [15]–[17]. In Figure 2c and 2d, SWI shows that there is a hemorrhage (within the yellow circle) in the left putamen (visible in the second and third MRI scans). We also observed that the veins around the stroke lesion in Figure 2c and 2d are darker than the contralateral normal side and also darker than those in the first time point (2b). This is because uncoupling between oxygen supply and demand within or around the stroke region causes a relative increase of deoxyhemoglobin levels.

A limitation of this approach is that we have to adjust the brain in the same location for all scans so that the vessels are in the same orientation for the different scans. Thus we can cancel the geometry dependent term (3cos^2^θ−1). If the position of the head is different by±5° relative to the main field, this will induce only a 2% error in the result. Another limitation is in the partial volume effect where the vessel may shift from being at the edge of a voxel to the center of a voxel and that can modify the phase in the vessel. In the former case the phase will be lower than that in the latter case. This effect however should be random and should average out over all the ten veins measured in the brain for any given time point. Finally, new methods such as susceptibility mapping may also provide a similar means to measure oxygen saturation in the future [18], but these methods are still under development and have their own artifacts and limitations.

## Conclusions

We have found that SWI provides a non-invasive method of measuring T2* changes in the brain and offers an alternative means of measuring changes in oxygenation levels in the brain. This capability complements the information available in the more standard imaging techniques of diffusion and perfusion. The results obtained by this study indicate that the oxygen saturation change may predict clinical outcomes for the stroke patient.
